# Supplementary material for: Fox dietary ecology as a tracer of human impact on Pleistocene ecosystems
Source: PLoS One. 2020 Jul 22;15(7):e0235692. doi: 10.1371/journal.pone.0235692 (PMC7375521; doi:10.1371/journal.pone.0235692)
Supplement: S1 Text — (PDF) [file pone.0235692.s001.pdf]

S1 Text:

Intra-individual variability

In order to investigate intra-individual variability, we examine each site separately. First we consider the archaeological assignment and then the morphological and palaeogenetical assignments to exclude multiple sampling of one individual. For the remaining ambiguous samples an intra-individual variation is only assumed for carbon isotopic values in a range of 0.6‰ and nitrogen isotopic values in a range of 0.7‰, according to DeNiro and Schoeniger (1). The results are additionally summarized in Tables S5. Table S6 shows the differences in carbon and nitrogen values of unsafe individuals. Values below the limits given by DeNiro and Schoeniger (1) are marked in red and indicate the same individual. Values above 1 show a clear difference and are marked in blue. It is very likely that there are two individuals, even if the second value is below the limit. Values between both limits are marked yellow.

Bockstein is a cave site complex from which we analyzed specimens from Bocksteinschmiede (BS; n = 5), Westloch (n = 1) and Bockstein-Törle (BT; n = 3). All samples came from different archaeological layers, except PLC-80 and PLC-84, which both came from BS III, but from different geological layers (GH 21 and GH 22, respectively). Due to the spatial and temporal distribution of the specimens, we do not assume multiple sampling of single individuals, and consequently strict inter-individual variability in the analyzed Bockstein samples.

In **Geißenklösterle**, two sets of samples each come from the same archaeological layer. There are two left fox tibiae from AH I (VLP-4 and VLP-5), which certainly belong to two different individuals. Furthermore, two lynx bones, one rib (PLC-18) and one mandible (PLC-19) from AH Ir were sampled, which could come from one individual. The range of isotope values suggests that both samples could belong to the same specimen.

In **Hohle Fels**, two sets of two wolf samples each originated from the same archaeological layers: JK 2180 (humerus) and JK2184 (metacarpal IV) came from AH IV and a genetic analysis (mtDNA), as well as direct dating could only be done with JK 2180 (publication in preparation; personal communication with Saskia Pfrengle), therefore, we cannot exclude that both samples could come from the same individual. However, the differences between the isotopic values make belonging to one individual quite unlikely (see Table S5). The two Gravettian samples JK 2174 (scapula) and JK 2183 (calcaneus) were taken from the related AH IIc and IIcf, but were both directly dated. The dates do not overlap, but are actually about 360 years apart (publication in preparation), indicating no intra-individual variability

Hohlenstein is another cave site complex, although we have only analyzed samples from Hohlenstein-Stadel. Most of the samples were found separately in space (find square) and time (Hieb), however, four samples show the same locality and time sequence. These are the two right wolf mandibles PLC-24 and PLC-25, and the red fox samples PLC-26 (mandible) and PLC-27 (humerus) from find square 19 and Hieb 2. While the wolf samples originate certainly from two different individuals, the fox samples may originate from the same specimen. An analysis of the mtDNA of both samples showed a strong relationship and does not exclude the possibility that it is one individual (personal communication with Yumeko Tarusawa). Since the stable isotope data are very comparable, we also assume that this could be corresponding to intra-individual variability.

The spatial position of the samples from **Sirgenstein** is unfortunately impossible to evaluate, since the cave was completely excavated by R.R. Schmidt in 1906 during a one-year excavation campaign and no find squares were recorded [2]. Consequently, it is also very difficult to exclude that only different

individuals have been sampled. In our study, this applies to red foxes, as we can only assume that we deal with at least four different individuals out of nine samples. PLC-66 to PLC-69 from the Aurignacian layer AH IV include a right and a left mandible, a left humerus and a left tibia. A study on mtDNA of PLC-67 and PLC-68 showed that these two samples (left mandible and humerus) are two individuals, the other two samples could not be analyzed genetically (personal communication with Yumeko Tarusawa). If we apply the genetic conclusion to the isospace, it is most likely that only PLC-66 and PLC-67 belong to the same individual. From the analyzed samples from layer AH I we can only assume that the two right mandibles PLC-43 and PLC-72 belong to two different individuals, and the two right humeri PLC-71 and PLC-73 are not the same individual. If we consider their position in the isospace, then PLC-43 and PLC-73 are very close to each other, which could indicate that they belong to the same individual. For the last three samples, PLC-71, PLC-72 and PLC-75, it is very unlikely that they originate from the same specimen due to the isotopic values.

Vogelherd Cave was excavated in a single action as well, namely in spring 1931 by G. Riek [3, 4]. Again, spatial localization of the samples is impossible, having sampled several bones of single individuals cannot be excluded. With the wolves, this refers mainly to four samples from AH IV: one radius (PLC-2), one tibia (PLC-45), one left and one right ulna (PLC-46 and PLC-47 respectively). Since no genetic studies or direct dating of these bones have been performed, it cannot be excluded that this is one single individual. However, the ranges of their isotopic values suggest an origination from two individuals (Table S5 and S6). The two Middle Paleolithic wolf samples from AH VIII, an astragalus (PLC-48) and a tibia (PLC-49) might belong to the same individual, but show large differences in their isotopic values. Therefore, it is more likely that they belong to separate individuals. Similarly, the two sampled Arctic foxes from AH IV, PLC-1 (tibia) and PLC-16 (mandible) are most likely single specimens, due to their isotopic values. Among the six red fox samples from AH IV, we can only be certain of four individuals, which were partially genetically determined. The mtDNA study suggests that PLC-9 (femur), PLC-11 (radius), and PLC-14 (right mandible) are not related and do not represent one individual. Furthermore, the three right mandibles (PLC-13 to PLC-15) are not from the same fox. Same applies to the two right tibiae, PLC-9 and PLC-10. Considering their position in the isospace, we could identify at least four individual red foxes: PLC-11 and PLC-15 could originate from one individual, PLC-9 and PLC-13 from a second and PLC-14 and PLC-10 represents the third and fourth individual.

In summary, we cannot exclude an impact of unrecognized same individual sampling among some analyzed bones on the inter-individual variability in our samples (S4 and S5 Figs). This concerns especially wolf and fox samples from the old excavations in Sirgenstein and Vogelherd. Although the samples from Aurignacian are the most affected (two cases in wolves and four cases in foxes), there is no mixing of the samples beyond the trophic niches (S4 Fig). This means that the calculations of trophic niches, as we identified in our study, are still correct.

References

1. DeNiro MJ, Schoeniger MJ. Stable carbon and nitrogen isotope ratios of bone collagen: variations within individuals, between sexes, and within populations raised on monotonous diets. Journal of Archaeological Science. 1983;10(3):199-203.
2. Schmidt RR, von Koken EFRK, Schliz JA. Die diluviale Vorzeit Deutschlands: E. Schweizerbart; 1912.
3. Riek G. Altsteinzeitliche Funde aus der Vogelherdhöhle bei Stetten ob Lontal. Schwäbisches Museum. 1932:1-9.

4. Riek G. Die Eiszeitjägerstation am Vogelherd im Lonetal. Tübingen: Akademische Verlagsbuchhandlung; 1934.
