# Supplementary material for: Fox dietary ecology as a tracer of human impact on Pleistocene ecosystems
Source: PLoS One. 2020 Jul 22;15(7):e0235692. doi: 10.1371/journal.pone.0235692 (PMC7375521; doi:10.1371/journal.pone.0235692)
Supplement: S2 Text — (PDF) [file pone.0235692.s002.pdf]

S2 Text:

Statistical test for isotopic variance of both fox species

To determine the extent to which the trophic niches of the two fox species overlap, it would be possible to use SIBER, similar to the trophic overlap of the large carnivores and high $\delta^{15}N$ foxes. However, we have been able to sample far fewer Arctic foxes (n = 11) than red foxes (n = 28). Divided over the three periods studied, the numerical differences are even greater (see Table 1). Therefore, we applied a Welch ANOVA test on $\delta^{13}C$ and $\delta^{15}N$ separated by the three periods by using JMP 14.2.0. This type of ANOVA tests for equality of mean values as well, but does not require the same sample size. The statistical test shows significant differences if the *p*-values is below 0.05.

Since we only have one Arctic fox in the Middle Palaeolithic, a statistical statement was not possible. However, this Arctic fox falls into the same niche as a red fox (low $\delta^{15}N$ foxes). In the Aurignacian, statistical analysis showed no differences between the $\delta^{13}C$ (F = 0.75, p = 0.40) and $\delta^{15}N$ values (F = 3.36, p = 0.09) of red and Arctic fox. Similarly, in the Gravettian, no difference was found between the $\delta^{13}C$ (F = 3.89, p = 0.10) and $\delta^{15}N$ values (F = 2.17, p = 0.19) of both species.
