# Supplementary material for: Fox dietary ecology as a tracer of human impact on Pleistocene ecosystems
Source: PLoS One. 2020 Jul 22;15(7):e0235692. doi: 10.1371/journal.pone.0235692 (PMC7375521; doi:10.1371/journal.pone.0235692)
Supplement: S3 Text — (PDF) [file pone.0235692.s003.pdf]

S3 Text:

Archaeological interpretation

Foxes from three sites were sampled for the Middle Palaeolithic: Bockstein and Hohlenstein-Stadel in the Lone Valley, and Hohle Fels in the Ach Valley (S1 Figure, Tab. 1). Conard (1) showed that the Swabian Jura was sparsely populated by Neanderthals in the Middle Palaeolithic, as suggested by the low density of lithic artefacts and burnt bones. Bones found from fetal horses and very young mammoths indicate that the caves were occupied primarily in winter and spring [2]. The main prey of the Neanderthals was a little different between both valleys. While in the Lone Valley horse was a common game species [3-5], in the Ach Valley mainly reindeer was hunted [2, 6, 7].

We will now compare these insights with the isotope data obtained from the foxes. During the Middle Palaeolithic of the Swabian Jura we found two different trophic niches of foxes. All of the high $\delta^{15}N$ foxes come from the Lone Valley. However, the two low $\delta^{15}N$ foxes come from both valleys. Since the population density of the Neanderthals in this region was low and the caves were only visited at certain times of the year, it is unlikely that the foxes were hunted by the Neanderthals. In addition, there is only one fox bone (phalanx from Bockstein, Krönneck (4)) with cut marks indicating that a fox was exploited by Neanderthals. In general, the use of small game such as hares, fish and birds was very limited during this period in both valleys [6]. Both trophic niches of the investigated foxes (high $\delta^{15}N$ foxes = commensal to large predators; low $\delta^{15}N$ foxes = rodent hunters) belong to the usual dietary spectrum of these small opportunistic predators.

For the Aurignacian, we have sampled foxes from three Lone Valley sites (Bockstein, Hohlestein-Stadel, Vogelherd) and three Ach Valley sites (Geißenklösterle, Hohle Fels, Sirgenstein; Fig. S2, Tab. 1). Compared to the Middle Palaeolithic, the human population density in the Aurignacian was about 10 to 15 times higher, as determined by Conard (1) based on artefact densities. The zooarchaeological record indicates that, as in the Middle Palaeolithic, reindeer and horse were among the most important game species of the Palaeolithic hunters [2-4, 6-10]. In addition, there are also a large number of mammoth remains found. In both valleys, these remains were bones (e.g., ribs) and ivory, which were further processed [2, 9, 11]. In contrast to the small herbivores, mammoths were not brought to the cave as a whole, but were butchered at the kill sites. Smaller herbivores, such as reindeer, were brought to the site in their entirety and butchered there [9]. There has been so far little research on seasonality in the Aurignacian, as well as in the Middle Palaeolithic. Difficulties in the interpretation of the seasonality are mainly due to the taphonomic processes in the layers, which additionally reduce the use of the few seasonality indicators [12]. Nevertheless, there are some studies for the Aurignacian. For the Ach Valley, this information is based on the evaluation of Geißenklösterle and Hohle Fels. On one hand the studies show a clear human occupation of the caves in winter and spring, based on cut marks on cave bear bones, the presence of fetal horse bones, and thick layers of burnt bones [2, 7]. On the other hand, a year-round occupation of the region could not be excluded, because of the increased evidence of the use of fish and birds, which were probably caught from spring to autumn (personal communication from Susanne Münzel; [6]). In the Lone Valley, Vogelherd and Hohlenstein-Stadel were the best investigated sites. For Vogelherd, Niven (8) assumed that the cave was occupied from late summer to late autumn due to reindeer migration, while for neighboring Hohlenstein-Stadel, Gamble (13) assumed that the cave was occupied in winter, spring and autumn. In contrast to the Middle Palaeolithic occupation of the Swabian Jura, it seems that during the Aurignacian, humans with a higher population density were present for longer times of the year.

In this context, we will now evaluate the trophic data obtained from the analyzed foxes. We have identified two trophic niches for the Aurignacian of the Swabian Jura. The high $\delta^{15}N$ foxes are the numerically strongest niche in the Aurignacian, as well as the intermediate $\delta^{15}N$ fox niche. Sites of both valleys are present in both niches, so that there is no regional division. From the zooarchaeological record we know that fox remains were significantly more abundant in the Aurignacian than in the preceding Middle Palaeolithic [6, 14] and perforated canines were used as pendants [3, 7, 15-22]. The use of foxes was obviously more important for people in the Aurignacian than in the Middle Palaeolithic. Furthermore, the cut marks found on mandibles [8] and long bones [6, 7, 18] show that foxes were butchered both for their fur and their meat. Both mandibles from the Aurignacian layer AH IV of Vogelherd [8] were sampled for this study (Arctic fox, Lab ID: PLC-16 and red fox, Lab ID: PLC-13). However, the niche and dietary reconstructions indicate that both specimens had different feeding strategies: one specimen (PLC-16) falls into the niches of high $\delta^{15}N$ foxes, while the other specimen (PLC-13) falls into the niche of intermediate $\delta^{15}N$ foxes. With this we have a clear proof that foxes from both trophic niches were exploited by humans. If we now compare the prey spectrum of humans, based on faunal evidence, and the average diet of the last years of life of foxes in the two niches, based on their stable carbon and nitrogen isotopes, we find some interesting overlap. With reference to the studies of Niven (9), we can try to locate the Aurignacian trophic niches in the environment. The high $\delta^{15}N$ foxes, as well as the large predators, show a high proportion of mammoth and other herbivores in their calculated diet (Fig. 4, Tab. 4). Mammoths could have died naturally in certain places [23, 24], or could have been hunted by humans [11, 25-27]. In both cases, scavenging carnivores would have easy access to their carcasses. Since the isotope signature in bone collagen reflects the average diet for several years [28-30], the $\delta^{15}N$ values obtained from the sampled animals cannot be explained by one-time events or seasonality. Therefore, it could be possible that large carnivores and foxes regularly visited the kill sites of Paleolithic hunters after they butchered their prey and took the necessary body parts with them. If one of the predators came too close to humans, they were also killed and (perhaps only partially) taken to the sites.
Besides rodents the main diet of the intermediate $\delta^{15}N$ foxes was reindeer. It is obvious that this diet was quite restricted, as it can be assumed that if a fox has the option to feed on carrion, it will not be choosy which carrion it is. The foxes from this trophic niche originate from three different sites in both valleys, thus excluding the possibility that this diet was a unique phenomenon. One explanation is that their diet came from the direct vicinity near the caves, as humans had only brought certain animals, such as reindeer, as complete bodies to the cave and processed them there [9]. In addition, foxes that live permanently close to humans are easier to hunt than those foxes that avoid humans.

Looking at the Gravettian period, we have included Geißenklösterle and Sirgenstein from the Ach Valley and Bockstein from the Lone Valley (Fig. S3, Tab. 1). The archaeological data, primarily based on lithic artefacts, show that the Gravettian is so far mainly traceable in the Ach Valley [31]. Radiocarbon dating of bones from Bockstein [31-33] and a worked 3rd incisor of a brown bear from Vogelherd [8, 34], however, suggests that at least sporadic settlement of the Lone Valley must have taken place at this time. The population density in the Gravettian of the Swabian Jura has not yet been investigated in detail. The most recent data were provided by Maier and Zimmermann (35), although their study provides only a generalized, broad overview of human population density in the Gravettian of Europe. They found that there was a strong decrease after the Aurignacian, although population densities could vary widely locally. However, the extent to which the sites investigated in our study were occupied by

humans cannot yet be fully determined. Most of the Gravettian foxes included in our study come from the AH I layer of Sirgenstein [10]. The results of the Sirgenstein faunal analysis show strong similarities with those found in Geißenklösterle and Hohle Fels and differ little from the Aurignacian [6, 7, 10]. Reindeer, horse and mammoth were identified as main prey, and small prey species such as hares, fish and birds became more numerous. We also see similarities to the Aurignacian in the distribution of the isotope analyzed foxes. Although the number of foxes found and analyzed is not as high as in the previous period, both share the same trophic niches: in the high $\delta^{15}N$ foxes all the sites we analyzed are represented, whereas in the intermediate $\delta^{15}N$ niche only foxes from Sirgenstein can be found. An indication that foxes were exploited by humans is found in two bones with cut marks from Hohle Fels [6] and further fox tooth pendants from Geißenklösterle and Hohle Fels [7, 15, 16, 18-22]. The conclusions we drew above for the Aurignacian can also be applied to the Gravettian. At least for Sirgenstein one strongly human-influenced trophic fox niche (intermediate $\delta^{15}N$ foxes) can be assumed. The high $\delta^{15}N$ foxes may also have fed without human influence, resulting in a commensalism to large predators (similar to the interpretation for the Middle Palaeolithic), although regular visits of human kill sites would be possible.

References

1. Conard NJ, Bolus M, Münzel SC. Middle Paleolithic land use, spatial organization and settlement intensity in the Swabian Jura, southwestern Germany. Quaternary International. 2012;247:236-45.
2. Münzel SC, Conard NJ. Change and continuity in subsistence during the Middle and Upper Palaeolithic in the Ach Valley of Swabia(south-west Germany). International Journal of Osteoarchaeology. 2004;14(34):225-43. doi: 10.1002/oa.758.
3. Kitagawa K. Exploring hominins and animals in the Swabian Jura: study of the Paleolithic fauna from Hohlenstein-Stadel. Unpublished Doctoral Dissertation: University of Tübingen2014.
4. Krönneck P. Die pleistozäne Makrofauna des Bocksteins (Lonetal–Schwäbische Alb). Ein neuer Ansatz zur Rekonstruktion der Paläoumwelt: Ph. D. Dissertation, University of Tübingen; 2012.
5. Krönneck P, Niven L, Uerpmann HP. Middle Palaeolithic subsistence in the Lone Valley (Swabian Alb, southern Germany). International Journal of Osteoarchaeology. 2004;14(3-4):212-24.
6. Conard NJ, Kitagawa K, Krönneck P, Böhme M, Münzel SC. The Importance of Fish, Fowl and Small Mammals in the Paleolithic Diet of the Swabian Jura, Southwestern Germany. Zooarchaeology and Modern Human Origins. Vertebrate Paleobiology and Paleoanthropology2013. p. 173-90.
7. Münzel SC. Die jungpleistozäne Großsäugerfauna aus dem Geißenklösterle. In: Conard NJ, Bolus M, Münzel SC, editors. Geißenklösterle: Chronostratigraphie, Paläoumwelt und Subsistenz im Mittel- und Jungpaläolithikum der Schwäbischen Alb. Tübingen: Kerns Verlag; 2019. p. 147-327.
8. Niven L. The Palaeolithic occupation of Vogelherd Cave: implications for the subsistence behavior of late Neanderthals and early modern humans: Kerns; 2006.
9. Niven L. From carcass to cave: large mammal exploitation during the Aurignacian at Vogelherd, Germany. Journal of Human Evolution. 2007;53(4):362-82.
10. Bertacchi A. Subsistence strategies and environmental change during the Middle and Upper Palaeolithic in the Swabian Jura (SW Germany): insights from Sirgenstein cave [Master's Thesis]: Universität Tübingen; 2017.
11. Münzel SC, Wolf S, Drucker DG, Conard NJ. The exploitation of mammoth in the Swabian Jura (SW-Germany) during the Aurignacian and Gravettian period. Quaternary International. 2017;445:184-99.
12. Lyman RL. Vertebrate taphonomy: Cambridge University Press; 1994.
13. Gamble C, editor Hunting strategies in the central European Palaeolithic. Proceedings of the Prehistoric Society; 1979: Cambridge University Press.

14. Baumann C, Wong GL, Starkovich BM, Münzel SC, Conard NJ. The role of foxes in the Palaeolithic economies of the Swabian Jura (Germany). Archaeological and Anthropological Sciences. in review.
15. Camarós E, Münzel SC, Cueto M, Rivals F, Conard NJ. The evolution of Paleolithic hominin–carnivore interaction written in teeth: Stories from the Swabian Jura (Germany). Journal of Archaeological Science: Reports. 2016;6:798-809. doi: 10.1016/j.jasrep.2015.11.010.
16. Kitagawa K, Krönneck P, Conard NJ, Münzel SC. Exploring cave use and exploitation among cave bears, carnivores and hominins in the Swabian Jura, Germany. Journal of taphonomy. 2012;10(3-4):439-61.
17. Wehrberger K. Der Löwenmensch vom Hohlenstein-Stadel. In: Floss H, Rouquerol N, editors. Das Aurignacien und die Anfänge der Kunst in Europa, Internationale Fachtagung Aurignac, 16-18 September 2005: Editions Musee-forum Aurignac; 2007. p. 331-44.
18. Hahn J. Die Geißenklösterle-Höhle im Achtal bei Blaubeuren. Baden-Württemberg L, editor. Stuttgart: Theiss Verlag; 1988.
19. Hahn J. Zur Funktion einer Aurignacien-Feuerstelle aus dem Geißenklösterle bei Blaubeuren. Fundberichte aus Baden-Württemberg. 1989;14:1-22.
20. Conard NJ. Eiszeitlicher Schmuck auf der Schwäbischen Alb. In: Köbl S, Conard NJ, editors. Eiszeitschmuck - Status und Schönheit. Museumsheft 6. Blaubeuren: Urgeschichtliches Museum; 2003. p. 15-50.
21. Hahn J. Eiszeitschmuck auf der Schwäbischen Alb: Süddt. Verlag-Ges.; 1992.
22. Langguth K, Malina M. Katalog der Ausstellung Eiszeitschmuck - Status und Schönheit. In: Köbl S, Conard NJ, editors. Eiszeitschmuck - Status und Schönheit. Museumsheft 6. Blaubeuren: Urgeschichtliches Museum; 2003. p. 93-128.
23. Pitulko V, Pavlova E, Basilyan A. Mass accumulations of mammoth (mammoth ‘graveyards’) with indications of past human activity in the northern Yana-Indighirka lowland, Arctic Siberia. Quaternary international. 2016;406:202-17.
24. Pitulko VV. The Berelekh quest: a review of forty years of research in the mammoth graveyard in northeast Siberia. Geoarchaeology. 2011;26(1):5-32.
25. Münzel S. The production of Upper Palaeolithic mammoth bone artifacts from southwestern Germany. The world of elephants Roma: Consiglio Nazionale delle Ricerche. 2001:448-54.
26. Münzel S, editor Seasonal hunting of mammoth in the Ach-Valley of the Swabian Jura. The world of elephants Proceedings of the first international congress Consiglio Nazionale delle Ricerche, Rome; 2001.
27. Nikolskiy P, Pitulko V. Evidence from the Yana Palaeolithic site, Arctic Siberia, yields clues to the riddle of mammoth hunting. Journal of Archaeological Science. 2013;40(12):4189-97.
28. Ambrose SH. Preparation and characterization of bone and tooth collagen for isotopic analysis. Journal of Archaeological Science. 1990;17(4):431-51. doi: https://doi.org/10.1016/0305-4403(90)90007-R.
29. Bocherens H, Drucker D. Trophic level isotopic enrichment of carbon and nitrogen in bone collagen: case studies from recent and ancient terrestrial ecosystems. International Journal of Osteoarchaeology. 2003;13(1-2):46-53. doi: 10.1002/oa.662.
30. Bocherens H, Fizet M, Mariotti A, Lange-Badre B, Vandermeersch B, Borel JP, et al. Isotopic biogeochemistry (13C,15N) of fossil vertebrate collagen: application to the study of a past food web including Neandertal man. Journal of Human Evolution. 1991;20(6):481-92. doi: https://doi.org/10.1016/0047-2484(91)90021-M.
31. Conard NJ, Bolus M. Radiocarbon dating the appearance of modern humans and timing of cultural innovations in Europe: new results and new challenges. Journal of human Evolution. 2003;44(3):331-71.
32. Hahn J. Aurignacien, das ältere Jungpaläolithikum in Mittel-und Osteuropa: Böhlau; 1977.

33. Hahn J. Eiszeitliche Jäger zwischen 35000 und 15000 Jahren vor heute. Urgeschichte in Baden-Württemberg Konrad Theiss Verlag, Stuttgart. 1983:273-330.
34. Conard NJ, Niven LB, Mueller K, Stuart AJ. The chronostratigraphy of the Upper Paleolithic deposits at Vogelherd. Mitteilungen der Gesellschaft für Urgeschichte. 2003;12:73-86.
35. Maier A, Zimmermann A. Populations headed south? The Gravettian from a palaeodemographic point of view. antiquity. 2017;91(357):573-88.
