# Supplementary material for: Fox dietary ecology as a tracer of human impact on Pleistocene ecosystems
Source: PLoS One. 2020 Jul 22;15(7):e0235692. doi: 10.1371/journal.pone.0235692 (PMC7375521; doi:10.1371/journal.pone.0235692)
Supplement: S4 Text — (PDF) [file pone.0235692.s004.pdf]

S4 Text:

Applying the hypothesis to other archaeological sites

To apply our hypothesis to other sites, two important requirements must be given. Firstly, foxes must be present in the zooarchaeological record and secondly, isotope data of herbivores and carnivores (from the region, at best even from the site), including foxes, must be available.

An important site where these data are available is the Czech open air site Předmostí I. Archaeological studies showed that this site was inhabited for a long time in the Gravettian [1]. Mammoth remains are the most common, which also often show cut marks [2]. However, the second most common species are wolves and arctic foxes. Reindeer and horse are far less common. Předmostí I is therefore also interpreted as a seasonal mammoth hunting site [1]. The high number of predators alone indicates a time slot that was free of humans, so that these animals could scavenge. Bocherens (3) published the stable carbon and nitrogen isotope values of among others large herbivores, wolves and also six arctic foxes from this site. The δ^{15}N values of these foxes are generally lower than other carnivores, although they also cluster together with individual wolves, wolverines and brown bears. Similar to the groups of the high δ^{15}N foxes from the Swabian Jura, the Předmostí I foxes seem to behave commensal to the local large carnivores. A special aspect of this site is the group of reindeer-consuming canids, which are considered as palaeo-dogs and were probably fed by humans [3-6]. The diet of the arctic foxes, on the other hand, indicates unrestricted scavenging. A direct synanthropic commensalism, as we suggested for the Swabian Jura early Upper Palaeolithic, is not visible in Předmostí I. This can be explained by the seasonal human occupation of the site and the resulting low continuity of such a trophic niche, but also by the occurrence of palaeo-dogs, which probably kept other predators, including foxes, away from humans.

Another site that matches the mentioned requirements is Buran-Kaya-III, a rock shelter located on the southern Crimea. The zooarchaeological record shows that especially in the layers 6-1 and 6-2 (both dated to 37,100 - 33,100 cal BP, [7]) many saiga antelopes (*Saiga tatarica*) were killed and butchered by early Upper Palaeolithic hunters [8, 9]. Based on this, Buran-Kaya-III was interpreted as a seasonal hunting camp, used only by humans during the annual migration of the saiga antelopes [8, 9]. Besides saiga antelopes, bones of foxes, at least one wolf and humans were also found and investigated in an isotope study [10]. Drucker (10) sampled among others five foxes (both red and arctic foxes) from both layers. The main difference was observed in the δ^{13}C values between the foxes from layer 6-1 and 6-2. In addition to bulk collagen, the two amino acids phenylalanine and glutamic acid were also analysed. It was shown that most of the foxes had been feeding on saiga antelope. Humans, on the other hand, had mammoth in their diet in addition to saiga antelopes. However, this diet did not apply to two foxes. They are also lower in their δ^{15}N values and fed on smaller prey such as hares [10]. If we now consider that it takes several years to generate δ^{15}N levels in bone collagen, most foxes should also have had several years of regular access to saiga antelope as a food resource. Similar to the reindeer in the Swabian Jura, the saiga antelope is a food resource for foxes, which at that time and in this site could mainly have been obtained by humans [8-10]. The adaptation of the foxes' trophic behavior to human food remains in Buran-Kaya-III also shows the influence of humans on the Pleistocene ecosystem in Eastern Europe.

If we take a look at the Magdalenian of the pre-Alpine region of Germany and Switzerland (about 16,300 to 14,000 cal BP [11]), we see that three different niches have also been identified, namely “fox niche”, “wolf niche” and “dog niche” [12]. At that time the Swabian Jura was not densely populated by humans, which is also reflected in the low density of finds at the archaeological sites [11, 13-18]. Due to the

differences in isospace before and after the LGM, these periods are not directly comparable [19]. However, it is possible to compare the composition of the trophic niches and their dietary reconstructions. One fox from Vogelherd was sampled for the Magdalenian, which fell into the "wolf niche", the equivalent of the high δ^{15}N foxes in the pre-LGM. As in the Middle Palaeolithic and the early Upper Palaeolithic, there was a niche with foxes, which were commensal to large predators. Into the Magdalenian "fox niche" there fell both a fox from the Geißenklösterle and a fox from Hohle Fels. This trophic niche would correspond to the low δ^{15}N foxes, as they fed mainly on small prey as well. These two niches do not seem to have been substantially influenced by humans in the Magdalenian either [12]. But this is different with the "dog niche", which contains a fox in addition to all dogs. This niche, however, only existed in the Swiss site Kesslerloch. In contrast to the Swabian Jura, the Magdalenian was more present in the Kesslerloch and humans seemed to have occupied this region more intensively [20].

References

1. Svoboda J, Ložek V, Svobodová H, Škrdla P. Předmostí after 110 years. Journal of Field archaeology. 1994;21(4):457-72.
2. Musil R. Palaeoenvironment at Gravettian sites in central Europe with emphasis on Moravia (Czech Republic). Quartär. 2010;57:95-123.
3. Bocherens H, Drucker DG, Germonpré M, Lázničková-Galetová M, Naito YI, Wissing C, et al. Reconstruction of the Gravettian food-web at Předmostí I using multi-isotopic tracking (13C, 15N, 34S) of bone collagen. Quaternary International. 2015;359-360:211-28. doi: 10.1016/j.quaint.2014.09.044.
4. Germonpré M, Lázničková-Galetová M, Sablin MV. Palaeolithic dog skulls at the Gravettian Předmostí site, the Czech Republic. Journal of Archaeological Science. 2012;39(1):184-202. doi: https://doi.org/10.1016/j.jas.2011.09.022.
5. Germonpré M, Lázničková-Galetová M, Losey RJ, Räikkönen J, Sablin MV. Large canids at the Gravettian Předmostí site, the Czech Republic: The mandible. Quaternary International. 2015;359-360:261-79. doi: https://doi.org/10.1016/j.quaint.2014.07.012.
6. Germonpré M, Losey R, Lázničková-Galetová M, Galeta P, Sablin MV, Latham K, et al. Spondylosis deformans in three large canids from the Gravettian Předmostí site: Comparison with other canid populations. International Journal of Paleopathology. 2016;15:83-91. doi: https://doi.org/10.1016/j.ijpp.2016.08.007.
7. Péan S, Puaud S, Crépin L, Prat S, Quiles A, Van Der Plicht J, et al. The Middle to Upper Paleolithic sequence of Buran-Kaya III (Crimea, Ukraine): new stratigraphic, paleoenvironmental, and chronological results. Radiocarbon. 2013;55(3):1454-69.
8. Crépin L, Péan S, Lázničková-Galetová M. Comportements de subsistance au Paléolithique supérieur en Crimée: analyse archéozoologique des couches 6-2, 6-1 et 5-2 de Buran-Kaya III. L'Anthropologie. 2014;118(5):584-98.
9. Lanoë FB, Péan S, Yanevich A. Saiga antelope hunting in Crimea at the Pleistocene–Holocene transition: the site of Buran-Kaya III Layer 4. Journal of Archaeological Science. 2015;54:270-8.
10. Drucker DG, Naito YI, Péan S, Prat S, Crépin L, Chikaraishi Y, et al. Isotopic analyses suggest mammoth and plant in the diet of the oldest anatomically modern humans from far southeast Europe. Scientific reports. 2017;7(1):1-10.
11. Taller A, Bolus M, Conard N. The Magdalenian of Hohle Fels Cave and the Resettlement of the Swabian Jura after the LGM. Modes de contacts et de déplacements au Paléolithique eurasiatique/Modes of contact and mobility during the Eurasian Palaeolithic ERAUL. 2014;140:383-99.
12. Baumann C, Starkovich BM, Drucker DG, Münzel SC, Conard NJ, Bocherens H. Dietary niche partitioning among Magdalenian canids in southwestern Germany and Switzerland Quaternary Science Reviews. 2020;227:106032.

13. Münzel SC. Die jungpleistozäne Großsäugerfauna aus dem Geißenklösterle. In: Conard NJ, Bolus M, Münzel SC, editors. Geißenklösterle: Chronostratigraphie, Paläoumwelt und Subsistenz im Mittel- und Jungpaläolithikum der Schwäbischen Alb. Tübingen: Kerns Verlag; 2019. p. 147-327.
14. Wong GL, Starkovich BM, Conard NJ. Human Subsistence and Environment during the Magdalenian at Langmahdhalde: Evidence from a new Rock Shelter in the Lone Valley, Southwest Germany. Mitteilungen der Gesellschaft für Urgeschichte. 2017;26:103.
15. Napierala H, Münzel SC, Conard NJ. Die Fauna des Magdalénien vom Hohle Fels. In: Taller A, editor. Das Magdalénien des Hohle Fels Chronologische Stellung, Lithische Technologie und Funktion der Rückenmesser. Tübingen: Kerns Verlag; 2014. p. 275-311.
16. Krönneck P. Die pleistozäne Makrofauna des Bocksteins (Lonetal–Schwäbische Alb). Ein neuer Ansatz zur Rekonstruktion der Paläoumwelt: Ph. D. Dissertation, University of Tübingen; 2012.
17. Kitagawa K. Exploring hominins and animals in the Swabian Jura: study of the Paleolithic fauna from Hohlenstein-Stadel. Unpublished Doctoral Dissertation: University of Tübingen2014.
18. Niven L. The Palaeolithic occupation of Vogelherd Cave: implications for the subsistence behavior of late Neanderthals and early modern humans: Kerns; 2006.
19. Stevens RE, Hedges REM. Carbon and nitrogen stable isotope analysis of northwest European horse bone and tooth collagen, 40,000BP–present: Palaeoclimatic interpretations. Quaternary Science Reviews. 2004;23(7-8):977-91. doi: 10.1016/j.quascirev.2003.06.024.
20. Napierala H. Die Tierknochen aus dem Kesslerloch: Neubearbeitung der paläolithischen Fauna: Baudepartement des Kantons Schaffhausen, Kantonsarchäologie Schaffhausen; 2008.
