# Supplementary material for: Fox dietary ecology as a tracer of human impact on Pleistocene ecosystems
Source: PLoS One. 2020 Jul 22;15(7):e0235692. doi: 10.1371/journal.pone.0235692 (PMC7375521; doi:10.1371/journal.pone.0235692)
Supplement: S5 Text — (PDF) [file pone.0235692.s005.pdf]

S5 Text:

Possible impact of prehistoric people on Pleistocene ecosystems

As we could show in this study, the trophic behavior of foxes and a possible human impact on the Pleistocene environments could be documented. However, foxes are not the only animals that can make this possible impact visible. Studies on mammoths and horses also show a change in trophic niches [1, 2]. It is hypothesized that due to hunting pressure on mammoths in the Gravettian of the Swabian Jura their natural dietary niche (plants with high $\delta^{15}N$ values [3]) could not be completely occupied by these animals. As a consequence, direct competition by horses increased [1]. Zooarchaeological studies showed that early Upper Palaeolithic humans in this region hunted mammoth as their main prey [4-9], besides reindeer as already mentioned. The hunting took place probably in spring, as bones with cut marks of mammoth calves were also found [7, 9, 10]. Assuming that the reproduction rate of mammoths was low, hunting calves over a longer period of time would lead to a dramatic population decrease, which in turn would give the horses the chance to occupy the mammoths' trophic niche [1]. Besides modern humans, Neanderthals have also hunted mammoths [2, 11, 12] and may have caused population changes in these animals. However, this assumption could not be confirmed until now. Niche changes of mammoths and the associated ecological stress have so far only been proven in the early Upper Palaeolithic corresponding to modern humans [1, 2]. One important factor may be the population density of Neanderthals compared to that of modern humans [13]. We can assume with our study the regular presence of mammoth carcasses in the Middle Palaeolithic environment of the Swabian Jura by dietary reconstruction of high $\delta^{15}N$ foxes and large carnivores. However, this did not seem to have a significant impact on the trophic niche of mammoths, or on the occupation of the mammoth trophic niche by another herbivorous species, such as horse. In contrast, from the early Upper Palaeolithic onwards, modern humans changed the occupation of trophic niches in the ecosystem, so that horses also benefited from food resources that had previously been used exclusively by mammoths [1, 2]. Finally, with the appearance of the new intermediate $\delta^{15}N$ niches of foxes, we see another indication of changes in the Pleistocene ecosystem that may have been caused by humans, where foxes directly benefit from anthropogenic subsidies left around human camps.

References

1. Drucker DG, Vercoutere C, Chiotti L, Nespoulet R, Crépin L, Conard NJ, et al. Tracking possible decline of woolly mammoth during the Gravettian in Dordogne (France) and the Ach Valley (Germany) using multi-isotope tracking (13C, 14C, 15N, 34S, 18O). Quaternary International. 2015;359:304-17.
2. Wißing C, Rougier H, Baumann C, Comeyne A, Crevecoeur I, Drucker DG, et al. Stable isotopes reveal patterns of diet and mobility in the last Neandertals and first modern humans in Europe. Scientific Reports. 2019;9(1). doi: 10.1038/s41598-019-41033-3.
3. Bocherens H. Isotopic biogeochemistry and the palaeoecology of the mammoth steppe fauna. Deinsea. 2003;9(1):57-76.
4. Niven L. The Palaeolithic occupation of Vogelherd Cave: implications for the subsistence behavior of late Neanderthals and early modern humans: Kerns; 2006.
5. Niven L. From carcass to cave: large mammal exploitation during the Aurignacian at Vogelherd, Germany. Journal of Human Evolution. 2007;53(4):362-82.
6. Münzel S. The production of Upper Palaeolithic mammoth bone artifacts from southwestern Germany. The world of elephants Roma: Consiglio Nazionale delle Ricerche. 2001:448-54.

7. Münzel S, editor Seasonal hunting of mammoth in the Ach-Valley of the Swabian Jura. The world of elephants Proceedings of the first international congress Consiglio Nazionale delle Ricerche, Rome; 2001.
8. Münzel SC, Wolf S, Drucker DG, Conard NJ. The exploitation of mammoth in the Swabian Jura (SW-Germany) during the Aurignacian and Gravettian period. Quaternary International. 2017;445:184-99.
9. Münzel SC. Die jungpleistozäne Großsäugerfauna aus dem Geißenklösterle. In: Conard NJ, Bolus M, Münzel SC, editors. Geißenklösterle: Chronostratigraphie, Paläoumwelt und Subsistenz im Mittel- und Jungpaläolithikum der Schwäbischen Alb. Tübingen: Kerns Verlag; 2019. p. 147-327.
10. Barth M, Conard NJ, Münzel S. Palaeolithic subsistence and organic technology in the Swabian Jura. BAR International Series. 2009;2040:5-20.
11. Wißing C, Rougier H, Crevecoeur I, Germonpré M, Naito YI, Semal P, et al. Isotopic evidence for dietary ecology of late Neandertals in North-Western Europe. Quaternary International. 2016;411:327-45. doi: 10.1016/j.quaint.2015.09.091.
12. Münzel SC, Conard NJ. Change and continuity in subsistence during the Middle and Upper Palaeolithic in the Ach Valley of Swabia(south-west Germany). International Journal of Osteoarchaeology. 2004;14(34):225-43. doi: 10.1002/oa.758.
13. Conard NJ, Bolus M, Münzel SC. Middle Paleolithic land use, spatial organization and settlement intensity in the Swabian Jura, southwestern Germany. Quaternary International. 2012;247:236-45.
