# Supplementary figures and images for: Fox dietary ecology as a tracer of human impact on Pleistocene ecosystems
Source: PLoS One. 2020 Jul 22;15(7):e0235692. doi: 10.1371/journal.pone.0235692 (PMC7375521; doi:10.1371/journal.pone.0235692)

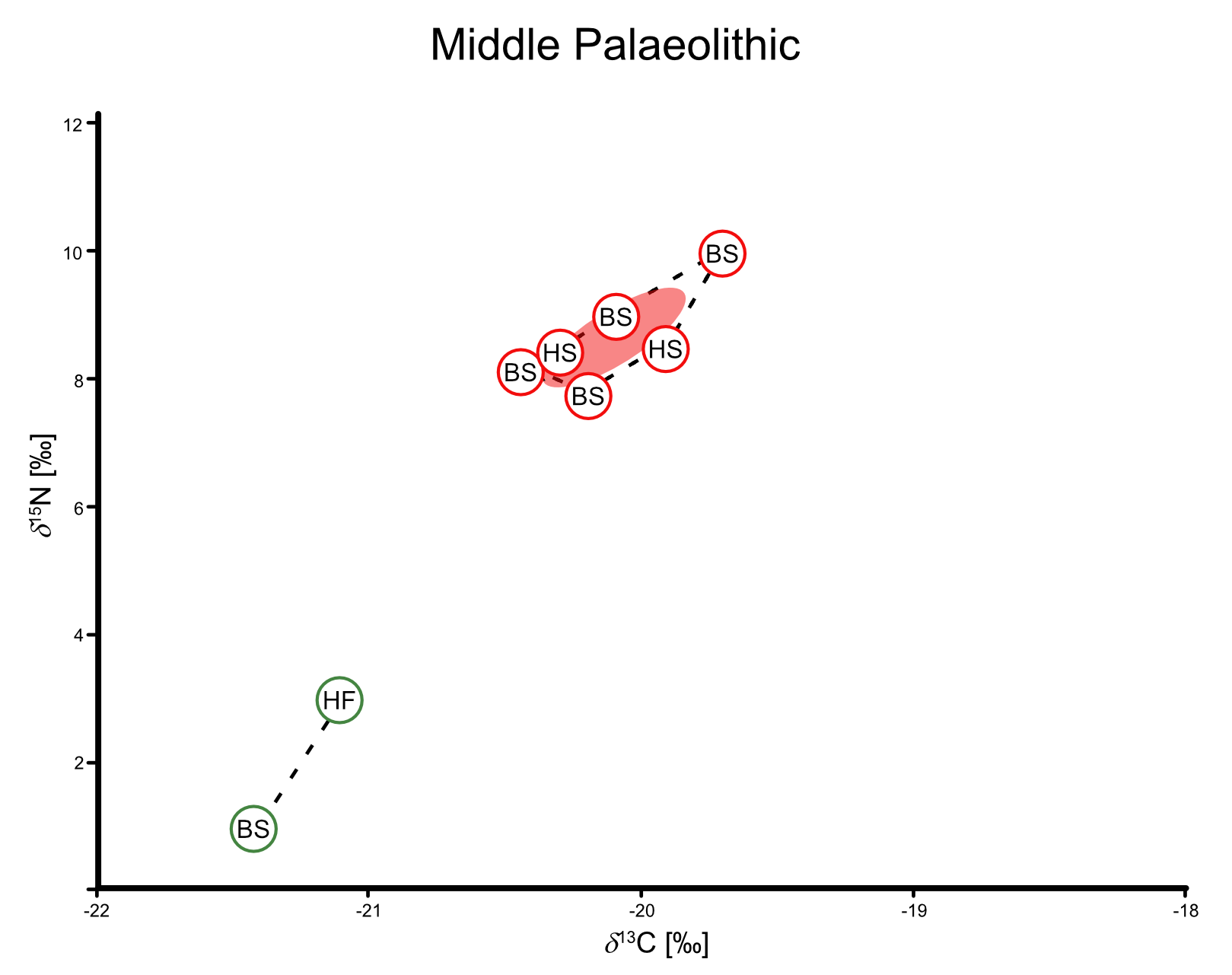

Supplement: S1 Fig — Dashed lines show the convex hull (outline of the niches), while the collard ellipses show the calculated Standard Ellipse Area (SEA) and reflect the core niches, based on Bayesian statistics. BS = Bockstein, HF = Hohle Fels, HS = Hohlenstein-Stadel. (TIFF) [file pone.0235692.s006.tiff]

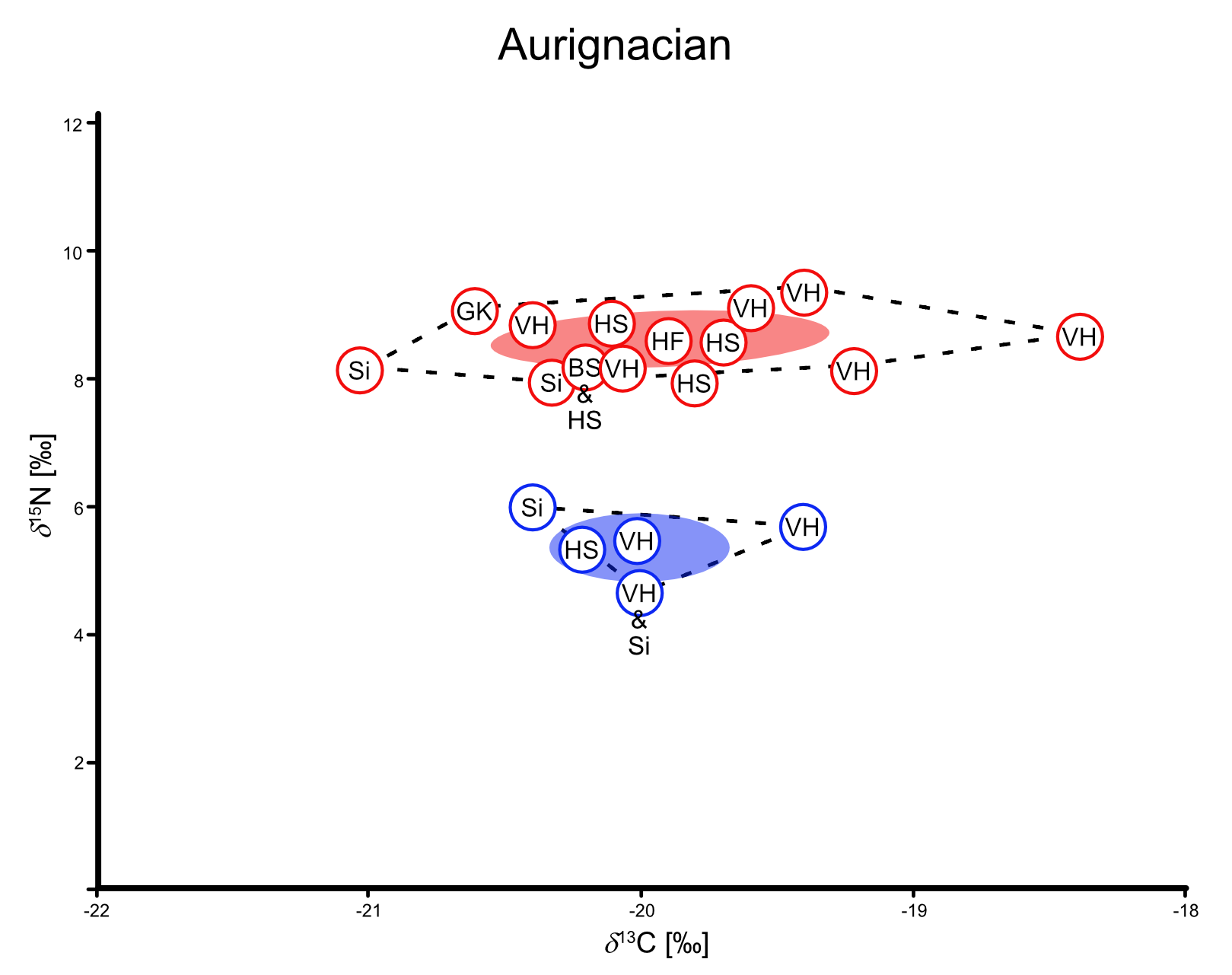

Supplement: S2 Fig — Dashed lines show the convex hull (outline of the niches), while the collard ellipses show the calculated Standard Ellipse Area (SEA) and reflect the core niches, based on Bayesian statistics. Red area = high δ15N foxes, blue area = intermediate δ15N foxes, BS = Bockstein, GK = Geißenklösterle, HF = Hohle Fels, HS = Hohlenstein-Stadel, Si = Sirgenstein, VH = Vogelherd. (TIFF) [file pone.0235692.s007.tiff]

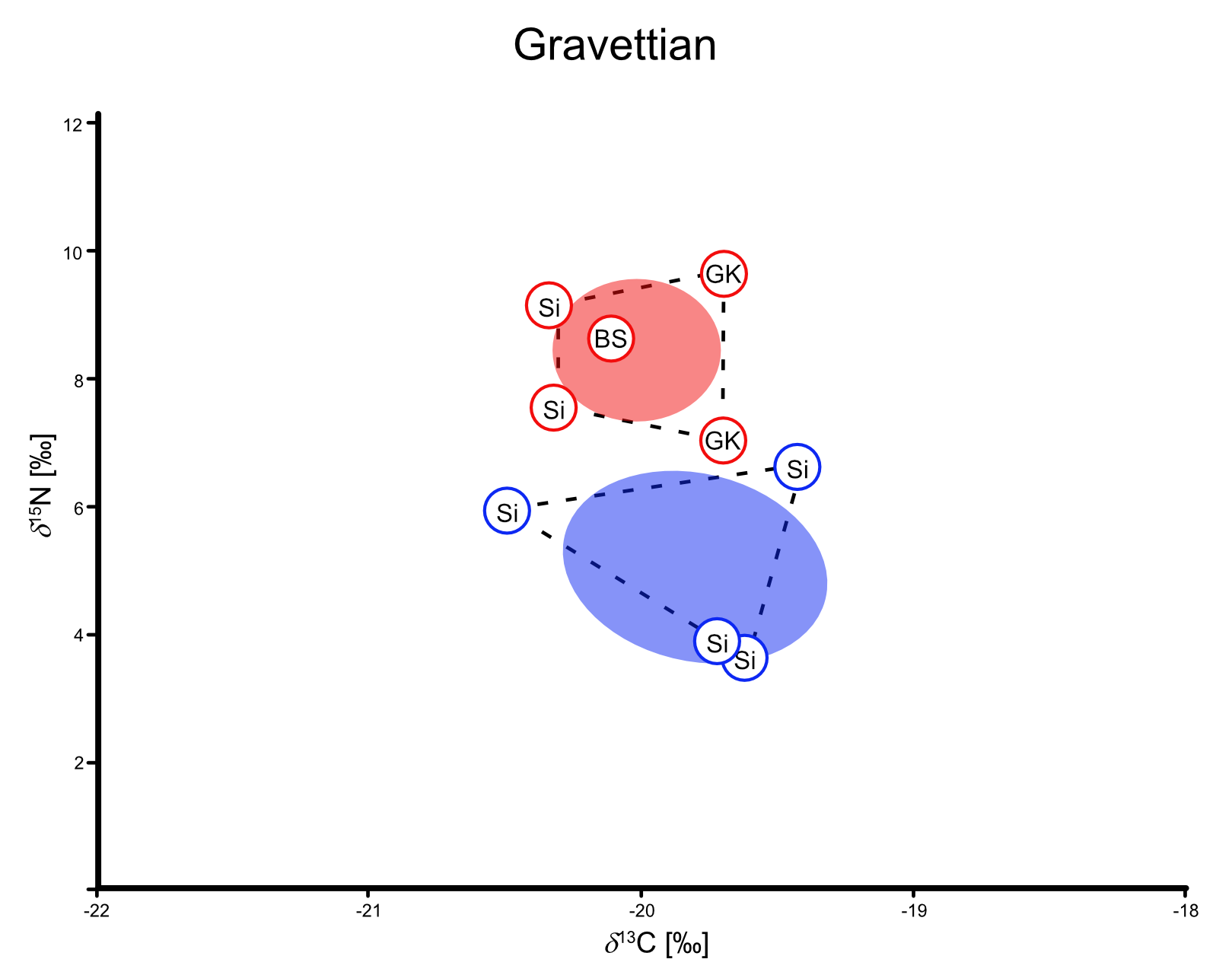

Supplement: S3 Fig — Dashed lines show the convex hull (outline of the niches), while the collard ellipses show the calculated Standard Ellipse Area (SEA) and reflect the core niches, based on Bayesian statistics. Red area = high δ15N foxes, blue area = intermediate δ15N foxes, BS = Bockstein, GK = Geißenklösterle, Si = Sirgenstein. (TIFF) [file pone.0235692.s008.tiff]

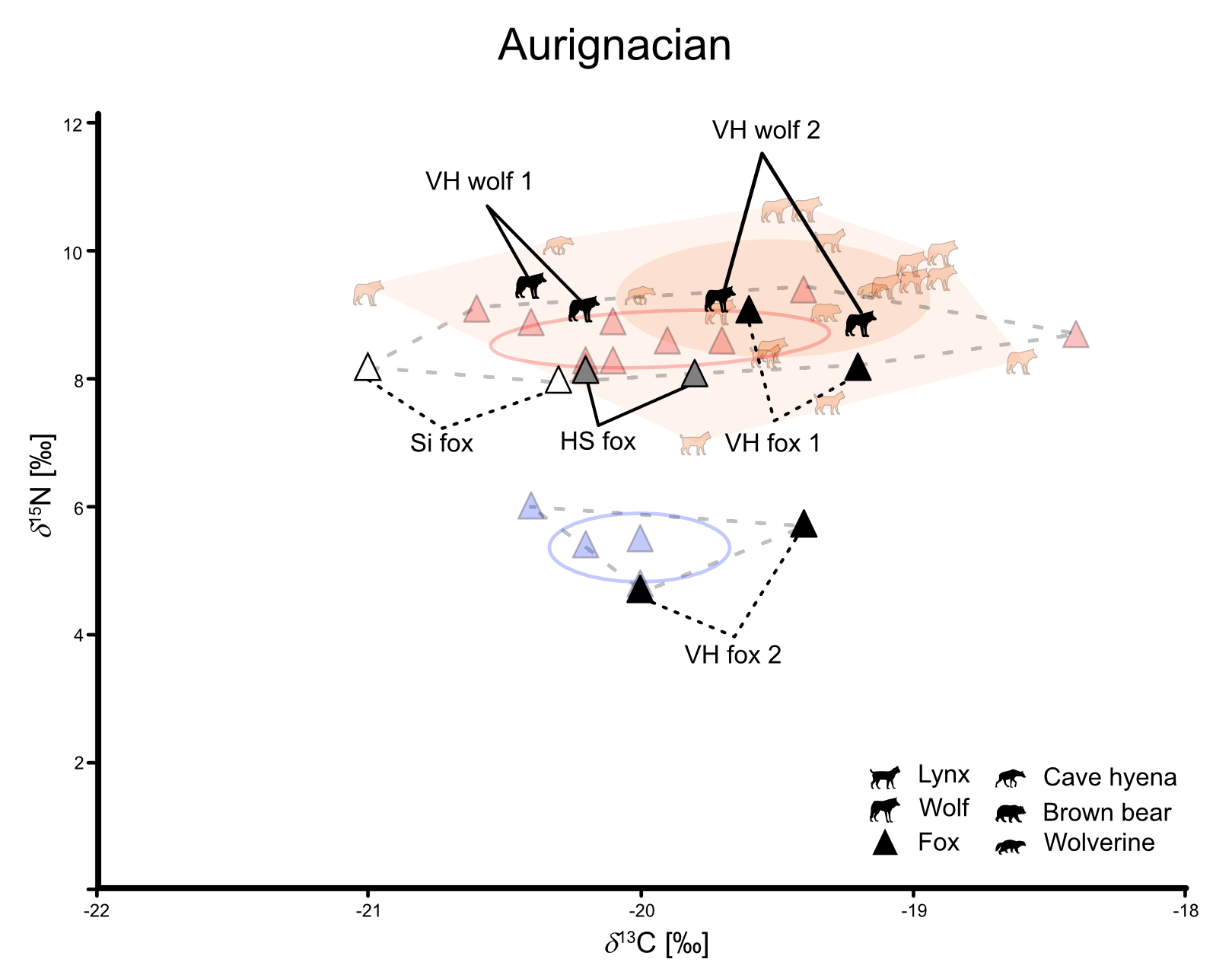

Supplement: S4 Fig — Black symbols show the potential affected samples from Vogelherd (VH), grey symbols show the potential affected samples from Hohlenstein-Stadel (HS) and white symbols show the potential affected samples from Sirgenstein (Si). Solid lines indicated most likely samples originated from one individual, according to the isotopic values and the limits. Dotted lines indicates a more unlikely origin from one specimen. (TIFF) [file pone.0235692.s009.tiff]

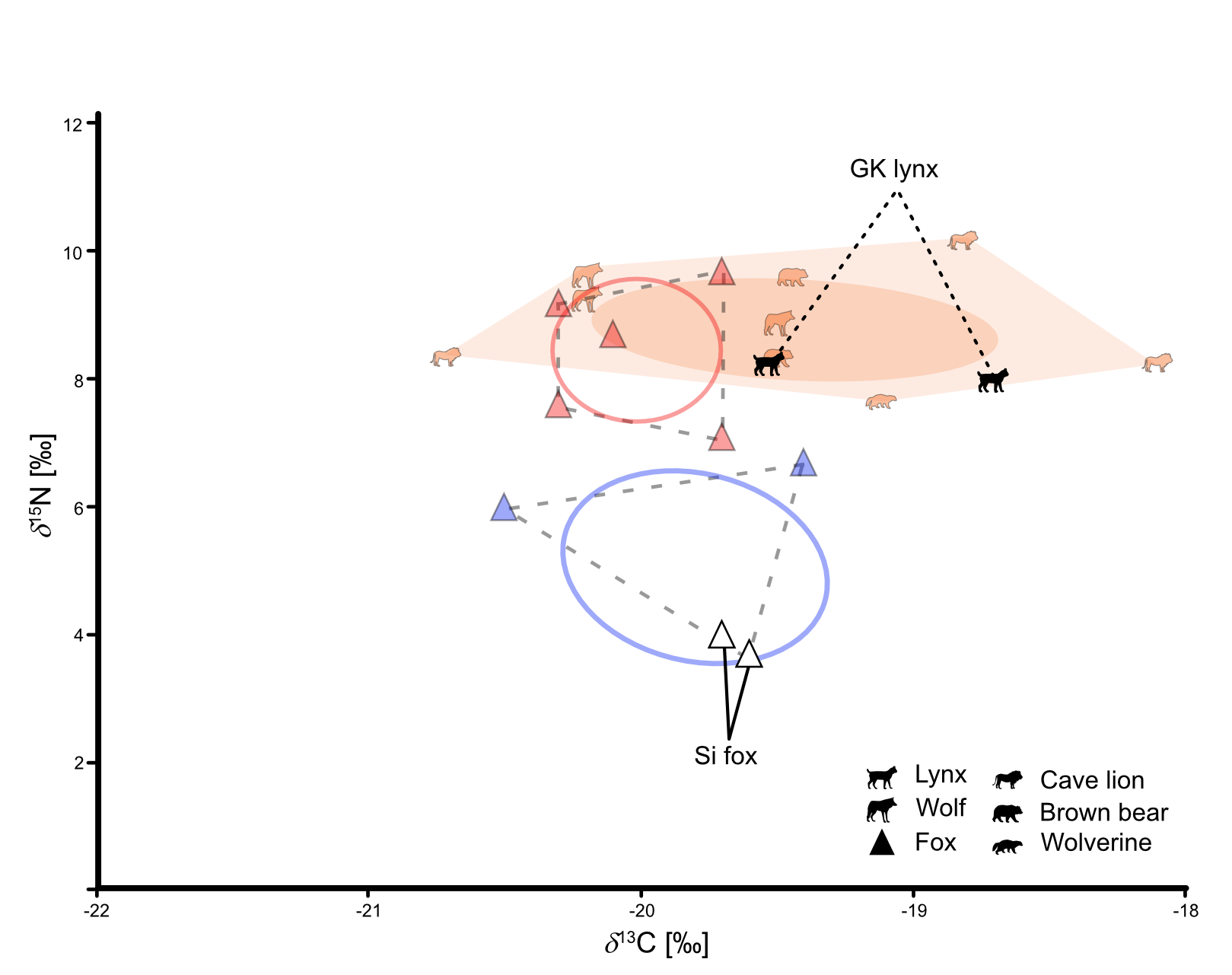

Supplement: S5 Fig — Black symbols show the potential affected samples from Geißenklösterle (GK) and white symbols show the potential affected samples from Sirgenstein (Si). Solid lines indicated most likely samples originated from one individual, according to the isotopic values and the limits. Dotted lines indicates a more unlikely origin from one specimen. (TIFF) [file pone.0235692.s010.tiff]
