# Supplementary material for: Fox dietary ecology as a tracer of human impact on Pleistocene ecosystems
Source: PLoS One. 2020 Jul 22;15(7):e0235692. doi: 10.1371/journal.pone.0235692 (PMC7375521; doi:10.1371/journal.pone.0235692)
Supplement: Attachment — Submitted filename: 00 Response to Reviewers (Version 3).docx [file pone.0235692.s017.docx]

**Response to Reviewers**

Dear Reviewers,

Thank you very much for the detailed reviews. We have made major changes in response to the comments. Firstly, the manuscript has been considerably shortened, especially in regard to the organization of the discussion. Information were kept by moving some chapters into the SI and using this opportunity to better structure them. In order to organize the manuscript more clearly, we have decided not to use the categories A, B and C, as this has may led to confusion as suggested from the comments. We now only refer to individual niches.

Detailed answers to Reviewer #1:

**It is unclear whether each specimen sampled can be confidently treated as a unique individual such that the resulting data represents strict inter-individual variability or it includes some intra-individual variability also. One example might be the two Canis lupus specimens PLC-37 and PLC-38 from the MPU archaeological horizon at the site of Hohlenstein-Stadel.**

Answer:
To examine this problem, we have written a separate SI chapter and referred to it in the main text. To summarize it briefly: We cannot completely exclude the possibility of multiple sampling of individual specimens, especially for the remains of the old excavations at Sirgenstein Cave and Vogelherd Cave. With reference to archaeological, morphological and genetic assignments, we were able, however, to confirm that most samples belonged to different individuals. When the methods mentioned above were not applicable, we examined the isotopic values. If they differed by more than 1‰, an affiliation to the same individual is excluded, whereas a difference of less than 0.7‰ suggests potential multiple sampling of the same individual. In summary, we determined a minimum individual number of 61 out of the 69 newly analyzed carnivore samples. More importantly, none of the potentially multiple sampled individuals affect the calculated niches since they clustered always into the same niche group. Thus, despite potential intra-individual variability, the number of calculated niches remains valid.

**Were all the sampled specimens mature (adult) individuals? If young individuals are included in the dataset it is important to remember that they will be isotopically enriched from the consumption of their mother’s milk and that this enrichment will last until the juvenile bone has been remodeled.**

Answer:
All sampled animals were adults, as it is now explicitly written in the main text.

**The discussion section is long-winded and could be significantly reduced in some sections. For example, the paragraph starting line 553 could easily be reduced to one or two sentences.**

Answer:
We have shortened the discussion considerably and structured it more comprehensibly.

**Make sure the in-text referencing follows the PLoS One guidelines. For example, I assume that “Bocherens, Drucker (54)” doesn’t follow the journal guidelines.**

Answer:
We have made these changes throughout the manuscript.

**Can you speculate as to why there are no low 15N foxes in the Upper Palaeolithic sites? Why might foxes have abandoned this ecological niche?**

Answer:
Foxes are very flexible in their feeding strategies, which means that they can change them accordingly to the food supply. We suspect that the resources provided by humans in the UP made it easier for foxes to scavenge the leftover carcasses than to actively hunt small mammals. The microfaunal record of the studied cave sites shows that the same rodents were present throughout the UP as were present in the MP, which thus rules out the climatic changes as the driving factor of the dietary behavior of fox over time.

**Line 22 – Can you make clear where the Swabian Jura is. This might not be obvious to some readers, including myself.**

Answer:
The Swabian Jura is a low mountain range in southwest Germany. We have now made this clearer in the manuscript and the map.

**Line 42 – Evidence for hominin hunting at ~3.5 Ma, as far as I am aware, is non-existent. The earliest evidences for hominin consumption of meat dates to perhaps ~2.5 Ma. Early meat consumption was likely achieved via scavenging or, if by hunting, was infrequent. The earliest real evidence we see of persistent hominin carnivory dates to ~2.0 Ma (for example, see Ferraro et al., 2013. PLoS One 8: e62174).**

Answer:
We have changed this too. Thank you for pointing that out.

**Line 53 – Do you mean “prehistoric”?**

Answer:
Yes, we have changed it.

**Line 56 – Again it would be great to state where Swabian Jura is in the introduction.**

Answer:
We have now made this clearer in the manuscript and the map.

**Line 106 – ranges**

Answer:
We have changed it.

**Line 118 – Only five (5) carnivore species are listed here: wolf, brown bear, red fox, and arctic fox.**

Answer:
In total, we considered seven carnivore species, but divided in the different periods. For the MP isospace, wolf, brown bear, red fox and arctic fox are included. For the Aurignacian isospace, wolf, wolverine, lynx, hyena, brown bear, red fox and arctic fox are included. For the Gravettian isospace, wolf, wolverine, lynx, cave lion, brown bear, red fox and arctic fox are included. We made this point clear in the manuscript.

**Line 231 – Were the same enrichment values applied to the other non-fox carnivores?**

Answer:
We did use the same enrichment factors, indeed, to be able to compare the diet evaluation on the same terms.

**Line 253 – As per the comment above, is it possible that these outliers are younger/older than other individuals in your sample?**

Answer:
No, because we used only adult specimens. The outliers in the rodents could indicate local environmental/climatic differences.

**Line 257 – The difference in 13C values between hare and arctic lemming is actually less than the difference between the two lemming species. Likewise, the difference between 15N of hare on the one hand, and lemming and voles on the other, isn’t significantly different – i.e., there is only a 0.2% difference between the greatest difference of hare vs. non-hare and non-hare vs. non-hare.**

Answer:
We have calculated the four groups of prey with a multivariable cluster analysis, based on their isotope values, not on the species affiliation. More detailed groupings might have been possible, but would have meant that the dietary calculation via MixSIAR would not have worked as accurately as it would have done with too many sources.

**Line 262 – I am curious as to how these groups were constructed? Just taking a quick glance at Fig. 2 it seems that many of the small mammals in the “horse group” could have easily been placed into the “small mammal group” (note the overlapping contour intervals).**

Answer:
We have calculated the four groups of prey with a multivariable cluster analysis, based on their isotope values, not on the species affiliation.

**Line 264 – These “species” groups are either not named after species (e.g., “rodent” group) or the named species is not the most common (e.g., “horse” group). For the latter, the most common taxon in the dataset is the genus Equus which includes horses, asses, and zebras. I recommend changing this to taxonomic groups, isotopic groups, or something else. Furthermore, as a reader I find these terms confusing for the dietary reconstruction (section 3.3). Particularly for the “horse” group which has almost an equal number of rodents. I wonder if there is a clearer and more informative system that could be employed?**

Answer:
For naming the groups, we picked out the taxon, which was the most common, which led to the fact that the group "Horse" also contains two lemming taxa (*Lemmus lemmus* n = 4, Dicrostonyx sp. n = 8). Horse, however, is the most common taxon (n = 19).

**Line 273 – It might be helpful to list the 15N ranges for each of the three niche categories. Also, under what rationale are the foxes grouped into the three nitrogen isotope groups? For example, why is PLC-73 with a 15N value of 3.7 included in the intermediate group and not the low group?**

Answer:
The grouping of foxes was also done by cluster analysis of nitrogen and carbon isotopic values and was performed separately for each period. As a result, not only the nitrogen isotopic ratios was taken into account in the niche reconstitution, but also the carbon isotopic ratios. However, we saw the biggest difference in the groups in nitrogen isotopic values and named the foxes after it. We made it clear in the manuscript.

**Line 275 – This line suggests that all niches (low, intermediate, and high 15N) are present in all three technological periods. I suggest rewording this.**

Answer:
We reworded the sentence that it is clearer now.

**Line 353 – I recommend changing the line “we conclude that they fed in a similar way”. There are huge** **differences in the feeding behavior of, say, foxes and hyenas.**

Answer:
We reworded the sentence that it is clearer now.

**Line 373 – Can you expand on the morphological study on the fox carnassial?**

Answer:
We have now gone into more details about this study in the manuscript.

**Line 379 – predators**

Answer:
We have changed it.

**Line 381 – This is a predicted diet not an average of the actual diet.**

Answer:
We reworded the sentence that it reads clearer now.

**Line 441 – I recommend rewording this sentence. Something along the lines of “From the zooarchaeological record we know that fox remains are significantly more abundant in the Auriganican than the preceding…….”**

Answer:
We reworded the sentence as suggested.

**Line 454 – The above comment about isotopic enrichment in young individuals is pertinent to assessing the validity of statements such as this.**

Answer:
We only sampled adults.

**Line 463 – What does a “one sided” diet mean?**

Answer:
It means unbalanced or restricted. We made this point clearer in the manuscript.

**Line 481 – How is the Gravettian, a specified technocomplex, identified as such by radiocarbon dating?**

Answer:
Yes, that was a little inappropriately phrased. We have now rewritten it so that it is clearer.

**Line 488 – Is this meant to say the “high 15N foxes”?**

Answer:
Yes, we lost the word, but now it has been changed.

**Line 495 – Is this reference to human kill sites? Because kill sites include predation by any animal (including humans).**

Answer:
Yes, it refers to human kill sites. We reworded the sentence that it reads better now.

**Line 526 – second?**

Answer:
The most important raw material for ornaments was ivory and fox teeth were the second most important. We have now rewritten it so that it is clearer.

**Line 532 – In the future it might be worth looking to strontium isotopes to test this hypothesis.**

Answer:
Yes, that's right. Thank you for this suggestion. We have included it in the section for further studies.

**Line 564 – What are the three niches of category A? Does this refer to the three archaeological periods (MP, Aurignacian, & Gravettian)? If so, can these be considered different niches just because they occur different times? Horses are considered to have occupied a similar niche for hundreds of thousands of years throughout the Plio-Pleistocene. I think revisiting how these different dietary and temporal niches are presented in the paper would be worthwhile. Lastly, I find this paragraph particularly difficult to follow and recommend rewriting it.**

Answer:
We have decided not to use the categories A, B and C, as this has may led to confusion. Therefore, we now only refer to individual niches in the manuscript.

**Line 586 – I’m not sure I buy the idea that large carnivores not being in the vicinity. At the very least, it seems likely that the home ranges of foxes in the vicinity of humans overlapped with some other larger carnivores in the broader region and, therefore, could have had access to larger carnivore refuse.**

Answer:
Our argument with the large carnivores, which were probably not tolerated by people in their vicinity, has been amended to clarify our line of reasoning. It now reads that isotopic results demonstrate that large carnivores did not feed regularly on the same resources as the intermediate *δ*^15^N foxes, namely prey hunted by human groups, which led us to conclude that the large predators were not tolerated by humans in the vicinity of their settlements, but foxes were.

**Line 645 – Are you able to present this graphically, perhaps in comparison to your own Swabian Jura data?**

Answer:
We have created a new table which compares the Swabian Jura with the other sites mentioned.

**Line 652 – “which also often show….”**

Answer:
We have changed it.

**Line 687 – What possible role did changes in climate play in restructuring ecology and altering large mammal tropic niches?**

Answer:
We have introduced a separate minor paragraph at the beginning of the discussion. Here we discuss the influence of climate and environmental changes on isospace.

Detailed answers to Reviewer #2:

**I may have missed it, but prior to combining the data from two fox species, do the authors confirm that the d13C and d15N values are statistically indistinguishable?**

Answer:
We did a statistical analysis in S2 Text, which led us to the conclusion that the two fox species did not show significant differences in their isotope values.

**Along the same lines, did the authors test for the effect of location among fox values within each time period? As well, most of the small mammal data are from a single site (HF), are other there data that support the use of these in dietary reconstruction for carnivores from other sites? Based on the map, the locations are not far apart, but I was curious if there is specific rationale for combining datasets across locations?**

Answer:
Which foxes come from which locality can be seen in the S1 - S3 figs. The small mammal data are mainly from Hohle Fels Cave (and two hares from Geißenklösterle Cave). As Reviewer #2 has already mentioned, the sites are quite close to each other and lithic compositions have shown that the people of Hohle Fels Cave either had contact with the groups in the other caves or were the same. We can therefore assume that the caves in the region were inhabited more or less simultaneously. The small mammals in the archaeological layers of the caves were almost exclusively from owls that hunted throughout the region. Therefore, these small mammals can be considered representative of all small mammals in the region. The large mammals used for food reconstruction also all originated from the sites discussed in the manuscript. We wanted to create a regional isospace that is more effective for the interpretation of foxes’ trophic behavior, as it reflects resources that foxes might have accessed to.

**Are the fox specimens (n=9) in the human-influenced dietary category B from horizons with evidence of human occupation? Wondering if there are other data to support a fox-human interaction for these individuals beyond just the estimated high proportion of reindeer in their diet? The fox-human interaction conclusion would be strengthened by information from multiple lines of evidence.**

Answer:
Yes, they do. We have only included sites and archaeological layers where human occupation has also been documented. This is also true for the other foxes. However, in archaeological layers it is never quite clear to what extent human action took place and over what time range. In a planned further paper, we will compare further (archaeological) proxies for the extent and intensity of human occupation and variations in population densities with the calculated niches of foxes. We will then be hopefully able to quantify the potential of the foxes as proxies of human activity with additional lines of archaeological evidences. However, this work is currently still in preparation.

**The paper is quite long, I found it a bit challenging to locate and extract information, and follow the authors’ arguments and chains of logic. Perhaps the manuscript could benefit from a more concise format.**

Answer:
The manuscript has been considerably shortened, especially in regard to the organization of the discussion. We thank both reviewers for their helpful remarks.

Sincerely yours,

Chris Baumann
